# RETRACTION: Long Noncoding RNASBF2‐AS1 Promotes Gastric Cancer Progression Via Regulating miR‐545/EMS1 Axis

**DOI:** 10.1155/bmri/9824751

**Published:** 2026-09-22

**Authors:** BioMed Research International

RETRACTION: M. He, L. Feng, L. Qi, M. Rao, and Y. Zhu. “Long Noncoding RNASBF2‐AS1 Promotes Gastric Cancer Progression via Regulating miR‐545/EMS1 Axis,” *BioMed Research International* 2020, no. 1 (2020): https://doi.org/10.1155/2020/6590303.

The above article, published online on 13 June 2020 in Wiley Online Library (wileyonlinelibrary.com), has been retracted by John Wiley & Sons Ltd. The retraction has been agreed after the authors initially requested retraction due to invalid results. An investigation into image duplication concerns raised by *Hoya camphorifolia* on PubPeer [1] has also been completed:

- Figure 2c shares unexpected similarities with Figure 3A in [2].
- Figure 2d shares unexpected similarities with Figure 2e in [3].

Separate attempts by the journal to contact the authors regarding these image concerns have not resulted in a reply. As a result, the data and conclusions of this article are considered unreliable.
